# Design of the Fall-Block Sensing of the Railway Line Pantograph Based on 3D Machine Vision Sensors

**DOI:** 10.3390/s18072305

**Published:** 2018-07-16

**Authors:** Kai Yang, Jianping Peng, Chaozhe Jiang, Xi Jiang, Longfei Xiao, Bangping Wang, Xiaorong Gao, Liming Xie, Hua Peng

**Affiliations:** 1 School of Physical Science and Technology, Southwest Jiaotong University, Chengdu 611756, China; yangkai@swjtu.edu.cn (K.Y.); adams.peng@swjtu.edu.cn (J.P.); longfei.xiao@my.swjtu.edu.cn (L.X.); gaoxiaorong@swjtu.edu.cn (X.G.); 2 School of Transportation and Logistics, Southwest Jiaotong University, Chengdu 611756, China; 3 State Key Lab of Rail Traffic Control and Safety, Beijing Jiaotong Unversity, Beijing 100044, China; xjiang@bjtu.edu.cn; 4 Chengdu Lead Science & Technology Co., Ltd., Chengdu 610091, China; wangpangping-y@sclead.com (B.W.); xieliming@sclead.com (L.X.); penghua-t@sclead.com (H.P.)

**Keywords:** 3D optical sensor, pantograph, surface defect, image processing

## Abstract

As an important part of the electric locomotive in railway transportation, the sensing and inspection of the pantograph has a significant effect on the safe operation of the train. In general, the pantograph carbon slip detection items include slide wear detection, slip strip crack detection, carbon slip fall-block detection and slip strip wear detection. The emergence and development of structured light measurement technology with 3D sensors provide new technical means for the acquisition of spatial 3D information. The three-dimensional data can not only obtain more information but also reduce the data deviation, thereby improving the measurement accuracy and work efficiency. At present, few studies have been conducted on the slide block and partial wear of the carbon slide. Therefore, this paper studies the detection of the pantograph slide block based on 3D sensor measurement technology. The experimental results indicate that it is feasible to adopt 3D measurement technology to detect the fall-block of the pantograph slide. In addition, a sound detection effect can also be obtained.

## 1. Introduction

In recent years, with the development of 3D sensor imaging technology and data processing technology, 3D vision technology has become the focus of the development of the computer field, which has attracted extensive attention and research [1,2,3]. The emergence and development of 3D laser measurement technology provide a new technical means for the acquisition of spatial 3D information, which can obtain more information about the shape of the measured object and improve measurement precision and accuracy [4,5]. Meanwhile, 3D data is more intuitionistic than 2D in visualization layout [6]. The development of calibration technology [7,8,9] and three-dimensional registration technology [10] has greatly promoted the application of three-dimensional measurement.

In terms of traditional traffic, specific key parts with mess data need to be monitored in detail, which requires more manpower or more artificial intelligence [11,12]. Original 2D information cannot meet the requirements of providing enough details for automatically evaluating by computer. For example, with the fast development of the high-speed train, railway mileage in China has maintained rapid growth. At the same time, China’s passenger and freight traffic is also increasing. The existing locomotives generally rely on the power system as traction power, in the system of which the pantograph serves as a key part. In order to ensure the safe operation of the train, it is extremely important to detect the state of the pantograph [11]. When the locomotive is running, it will inevitably produce sliding friction between the pantograph and the catenary. Under normal conditions, the wear of the carbon slide plate is more uniform and it will be changed when the carbon slide wears to its limit [11]. However, since each operation line between the electric bow and the contact wire under the flow condition of different carbon slides will be torn during use, due to the abnormalities, such as stripping off the block, crack, abrasion and other abnormal problems, the carbon slide life will be significantly reduced, which may affect the normal operation of the train. The carbon slide block, a lack of block and the partial wear phenomenon are mainly due to some mechanical reasons or the impact caused by the impact of both sides generated by the uneven force of the carbon slide plate when the train is in operation. The dynamic detection equipment of the pantograph has been widely applied in the railway field in China and it is extremely important to ensure the safe operation of the railway locomotive. However, at present, most equipment is employed to detect the pantograph sliding wear value and the pantograph tilt angle. According to railway overhaul rules in China, the pantograph slide is required to be inspected, and this is usually conducted by manpower. Normal 2D sensors do not have the ability to find the drop block and the attitude. Therefore, in this paper, research on pantograph slide block detection based on 3D sensor technology is carried out with the attitude through further information.

## 2. Pantograph Detecting Method Review

Research shows [11] that China has spent a lot of manpower and material resources carrying out pantograph detection system research and development. Through the efforts of the scientific research personnel, the pantograph detecting system, from the most primitive detection by maintenance staff to the installation of optical fiber type detection on the electric locomotive, the pantograph detecting system based on the triangulation method has been realized [13]. Through practical experience, it can be concluded that there are certain defects in the above detection methods, such as the need for power failure and parking, high cost of detection, low detection accuracy and low efficiency.

### 2.1. The Traditional Detection Method

**Figure 1:**
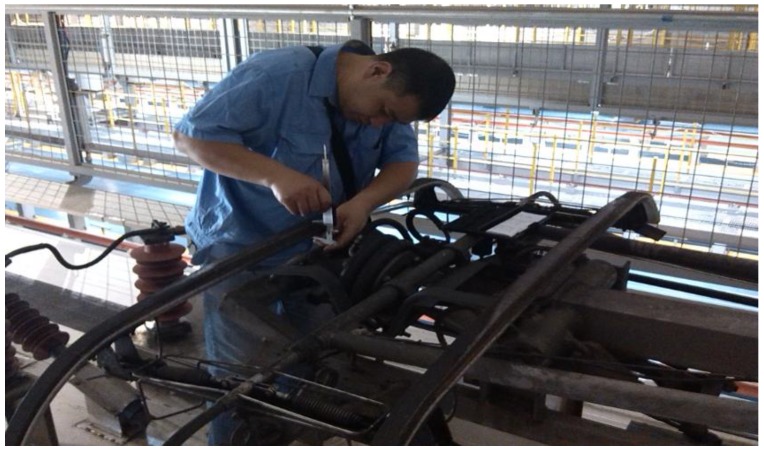
Traditional detection method.

Traditional detection of the pantograph needs the locomotive to be driven into the depots and parks and bowed down with the power off. Subsequently, maintenance staff on the roof inspected the pantograph for abnormal wear and special tools are used to measure abrasion of the pantograph [14]. Figure 1 presents the traditional detection method. At present, in the domestic operation of the railway line, this traditional method of detection is still one of the main methods. However, because of its low efficiency, big workload and low accuracy, there is little measurement. The method cannot adapt to the rapid development of railway transportation.

### 2.2. The Online Intelligent Detection System

**Figure 2:**
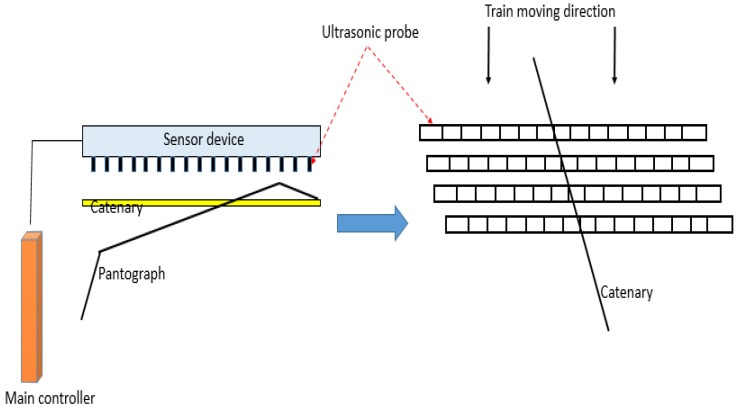
The principle of the ultrasonic measuring device.

In Japan, the East Japan Railway Company developed the pantograph slide abrasion automatic measuring device and the schematic of its principle is as follows (Figure 2). In the device, sensors are adopted to project waves to the object through the air and then reflected waves are sent back to the sensor. According to the transmission time of the ultrasonic wave and the wave velocity passing at that time, the surface thickness of the skateboard was obtained after calculation and the concave and the groove of the skateboard’s surface were detected [15]. In addition, the device adopts the ultrasonic ranging principle.

**Figure 3:**
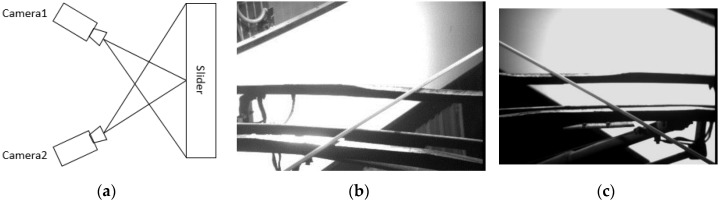
(**a**)The principle of the monitoring system; (**b**) the left picture captured by the left camera; (**c**) the right picture captured by the right camera.

In China, Southwest Jiaotong University has developed the electric bow and the roof condition dynamic monitoring system [14]. This system can be used to detect the abrasion of the slide, which can describe the wear curve for the reference of maintenance personnel. The main feature of this system is that it is installed on the locomotive warehousing line with high efficiency and low speed. Besides, it mainly uses the six charged couple device (CCD) cameras with flash lights, including 2 sets used to get centerline deflection and the remaining 4 sets used for slide abrasion with image processing. For the slide detection cameras shown in Figure 3, each camera captures more than half of the pantograph slide. However, since 3D information of the bow is unavailable, it cannot get the calculation result of the missing block.

A method for non-contact measurement of relevant geometric parameters has been proposed by applying hardware devices such as laser scanners, photoelectric encoders and industrial controller programming [16]. Yin Baolai et al. [17] proposed the use of ultrasonic sensors as detection elements to detect pantograph abrasion. In Reference [18], a system for visually measuring the pantograph slide wear of monorail trains using line structured light on the wear of the pantograph skateboards for monorail trains has been proposed. The image analysis is employed on the pantograph profile light fringe images acquired by industrial cameras. The calculation of the wear value of the slide plate is performed and the real-time operating state of the pantograph is estimated according to the wear value. Then, the information is comprehensively analyzed and processed to eliminate pantograph failure.

The non-contact distance measurement has high detection efficiency and small disturbance of driving but the laser detection is featured with a relatively single function and the ultrasonic detection accuracy is relatively poor.

Another Japanese Railway Company [19] has developed a pantograph automatic testing device that adopts the six sets of CCD industrial camera in the running locomotive pantograph, including 2 sets used for observation of the pantograph and the rest 4 sets used for image processing. The residual thickness of the skateboard was measured by the image processing and the abnormal phenomena such as the deformation of the bow, the gap of the bow and the wear.

The main detection method currently applied to the pantograph still depends on the 2D camera, that is, the in-plane measurement is performed by extracting the characteristics of the measured object in the grayscale image. However, this method cannot measure information such as height, depth and thickness in the *Z* direction.

In recent years, with the rapid development of three-dimensional image capturing technology and data processing technology, 3D vision technology has become the focus of development in the field of computer vision [15]. The 3D vision system starts from the image acquired by the camera and calculates the geometric information such as the position and the shape of the three-dimensional environmental object, thereby reconstructing and recognizing the object in the environment. Mara Cristina and Silvia Logozzo et al. use 3D optical scanning technology to make wear assessment on biomedicine and industry, which get significant advantage compared with other wear evaluation methods [20]. The emergence and development of 3D laser measurement technology has provided an entirely new technical means for the acquisition of spatial 3D information. The added third dimensional data can not only acquire more information on detected objects but also reduce data deviation, improve measurement accuracy and increase work efficiency. Consequently, a method for detecting the fall-block of the pantograph by using the three-dimensional detection technology of structured light is proposed in this paper.

## 3. The Principle of Structured Light 3D Detection

**Figure 4:**
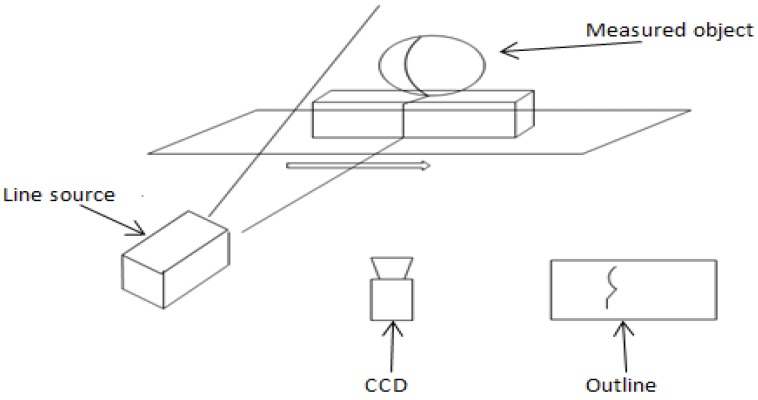
The Principle of Structured Light 3D Detection.

The basic idea of structured light 3D detection technology is to use the geometric information of the lighting source to extract the geometric information in the scene. The light stripe is generated on the surface of the object by using a light plane and the fringes are detected in the images considering the shape and the discontinuity of the stripes. Based on the geometry of the light source, the structured light includes spots, stripes and meshes. This method has the advantages of reduction of the calculation complexity, high speed of scanning and the high precision of measurement [4]. It is especially suitable for the indoor environment, where the surface reflection of the object is relatively good. The principle of structured light 3D detection is presented in Figure 4.

**Figure 5:**
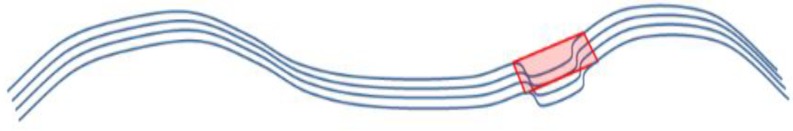
The Fall-block of the Slider.

The fall-block of the slider is detected by the change of the top surface of the larger skateboard which can be seen in Figure 5. The average level of the top surface is obtained by averaging all the laser profiles along the top in the X direction. Then, the resulting outline is adopted to determine the space gap in the contour. A lack of volume exceeding the preset threshold will be reported for defects.

## 4. The 3D Detection System Based on the 3D Sensor

**Figure 6:**
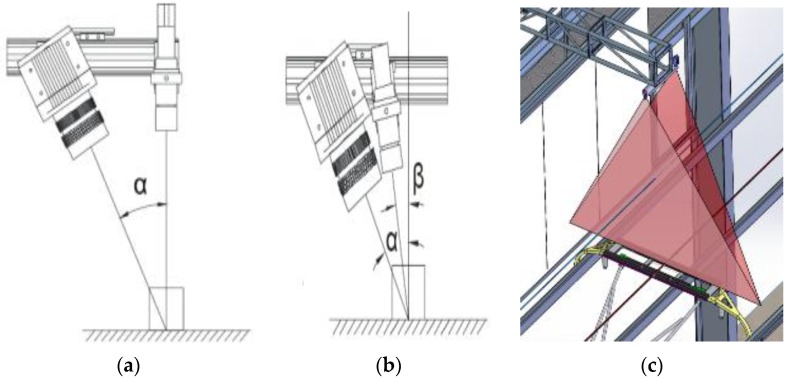
(**a**) System Layout 1; (**b**) System Layout 2; (**c**) The Mechanical Drawing Diagram.

The 3D sensor adopted is based on the principle of line structure light 3D detection. Because of its high precision, high speed and multiple scanning, it has been widely used for rapid measurement of industrial machine vision in 3D. At present, two layouts are widely applied in 3D measurement, as shown in Figure 6.

As shown in Figure 6a, the laser is perpendicular to the incident and the camera is oblique to the upper surface of the pantograph slider. The precision relationship between the *Z* direction and the *X* direction can be expressed as Function (1). The advantages of the layout (**a**) are that the height of the *Z* direction is simple and the high resolution of *Z* can be obtained. Besides, it is easy to be calibrated. The disadvantage is that the installation requirements of the device are relatively high and thus the actual thickness of the pantograph slide cannot be obtained. Only through detecting the change of the height of the surface can the excessive wear be determined.

(1) ΔZ≈ΔX/sin(α)

Figure 6b shows the laser oblique incidence and the camera oblique shot. In this layout, the upper surface of the sliding plate and the front surface can be captured by the camera. By finding the lower edge and the upper edge of the front surface of the bow, the wear and the resolution in the *Z* direction are determined using the height difference of the two positions and the laser tilt angle. The advantage of the layout (**b**) is that it can get the actual thickness of the pantograph slider and the disadvantages are that the calculation difficulty is increased in the *Z* direction. Besides, the precision is lower and the light intensity is weak. The precision relationship between the *Z* direction and the *X* direction can be written as Function (2).

(2) ΔZ≈ΔX×sin(α)/sin(α−β)

## 5. Verification of Key Parameters

### 5.1. Shooting Height and Laser Line-Width

**Figure 7:**
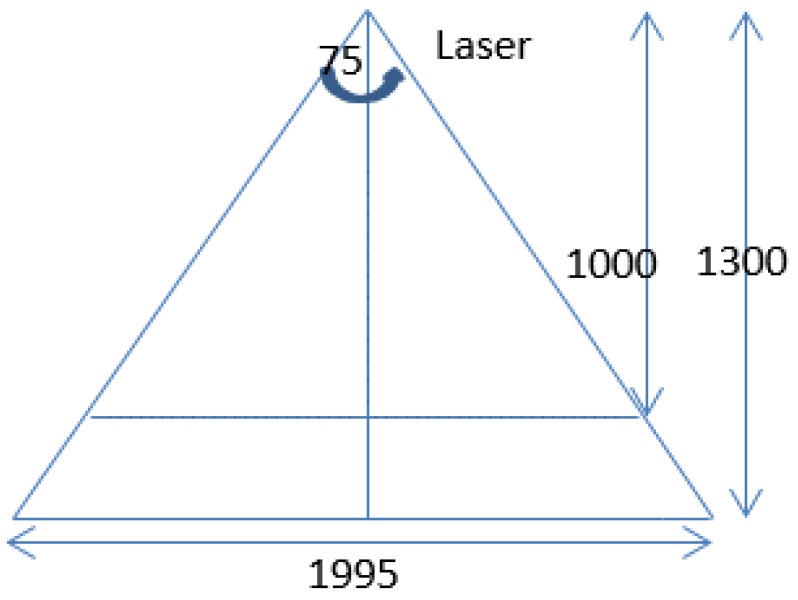
Structure Diagram.

The laser divergence angle is 75 degrees and the width of the pantograph in the workshop is 1450 mm. The required parameters can be calculated according to Figure 7. If the laser line is used to cover the whole pantograph, the height of the surface of the laser hole should be at least 945 mm. The height is set to 1000 mm and the height of the pantograph itself is 300 mm. The height of the camera should be in the range of 1000 mm–1300 mm and the nearest distance and the longest distance of the laser line are 1534.5 mm and 1995 mm respectively, which can satisfy the requirements of the pantograph detection.

### 5.2. Camera FOV

The 3D camera has a resolution of 1536 × 512 and a pixel size of 9.5 μm × 9.5 μm. The direction of the chip is parallel to the pantograph. If the focal length of the lens is 8 mm, the width of the field of the view angle is calculated as 34 degrees in accordance with Function (3). If the focal lengths are 12 mm, 16 mm and 25 mm, the corresponding lens field angles are 23 degrees, 17 degrees and 11 degrees accordingly.

(3) θ=atan(A/500×C/f)×180/π×2 

where A is the camera resolution, C is the pixel size and f is the focal length of the camera.

### 5.3. Camera Field Width

**Figure 8:**
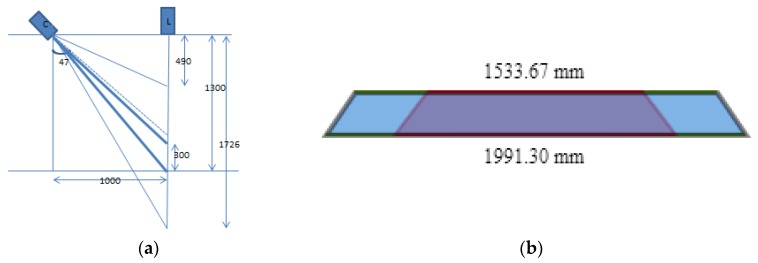
(**a**) The Sketch Map of the Optical Layout; (**b**) The Camera Field.

Suppose the angle of the axis camera and the laser center axis is 47 degrees, the focal length of the lens is 8 mm and the distance between the laser and the camera is 1000 mm. In this case, the minimum height of the laser line in the full view is 490 mm and the maximum height is 1726 mm. The height of the shooting range of 1000–1300 mm is taken into account which comply with this range. The vertical direction on the surface of the camera and the laser surface position are shown in Figure 8.

## 6. Experimental Analysis

In the current experiment, a 3D sensor was chosen which can collect up to 35,000 profile information every second, each section of which contains 1536 high-quality 3D coordinate points. The 3D data can be transmitted directly to the computer via a Gigabit Ethernet interface. With an infrared filter at the same time, the ambient light interference becomes smaller.

**Figure 9:**
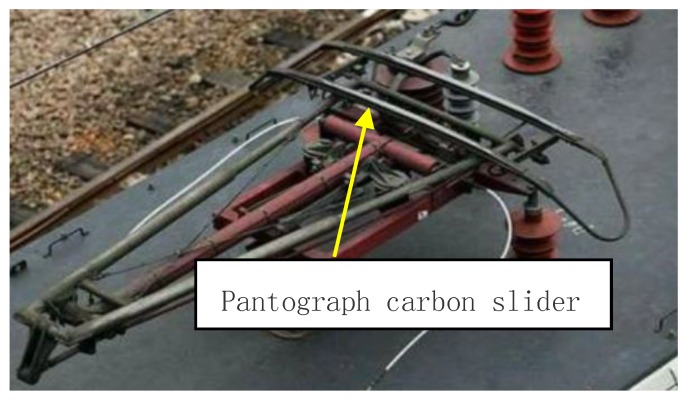
Pantograph.

According to the verification, in the case of Layout a, when the focal length of the lens f is 12 mm, the lens aperture is 1.4 and the number of the opened window line is 70. The highest resolution on the *Z* direction can be obtained. Figure 9 presents the pantograph.

**Figure 10:**
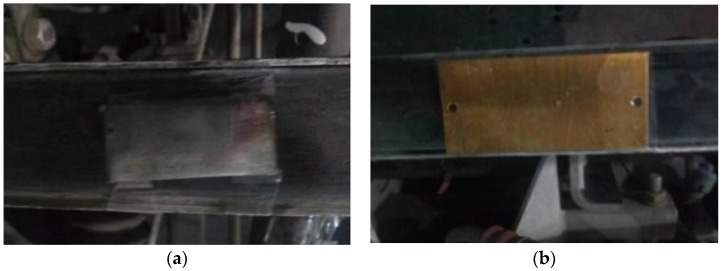
(**a**)Measured Object 1; (**b**) Measured Object 2.

In order to verify the feasibility and precision, the two objects of different heights on the pantograph are shown in Figure 10.

**Figure 11:**
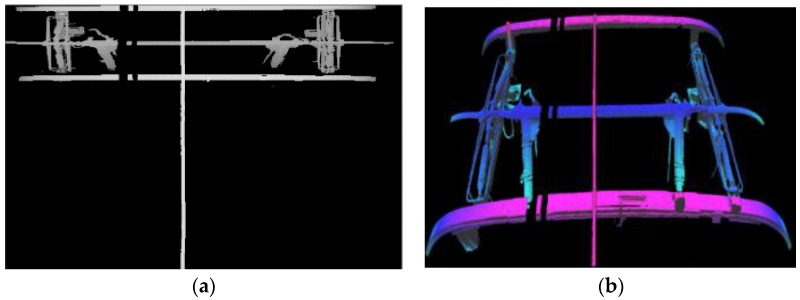
(**a**) Experiment 2D Data; (**b**) Experiment 3D Data.

The pantograph 2D and 3D data are shown in Figure 11.

**Figure 12:**
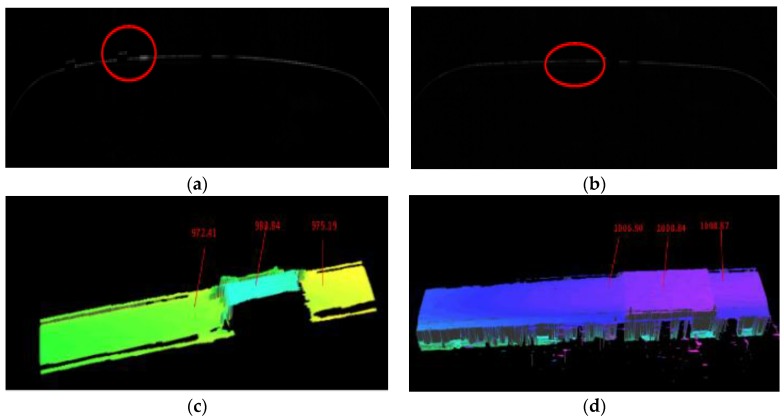
(**a**) 2D Data of Object 1; (**b**) 2D Data of Object 2; (**c**) 3D Data of Object 1; (**d**) 3D Data of Object 2.

The actual height of Object 1 is 12.9 mm and that of Object 2 is 0.9 mm. The laser line graph can be obtained respectively as Figure 12. According to the corresponding average resolution, the experiment value of Object 1 is calculated as 13.08 mm and the error is 0.18 mm. The experiment value of Object 2 is 1.09 mm and the error is 0.19 mm, which is within acceptable limits.

The results show that the error of Object 1 is larger. The main reasons include two parts. One is the calibration error and the other is the artificial errors caused by 3D data selection of the point’s location. However, based on the experiment results, an object of about 1 mm from 3D data can be identified.

**Figure 13:**
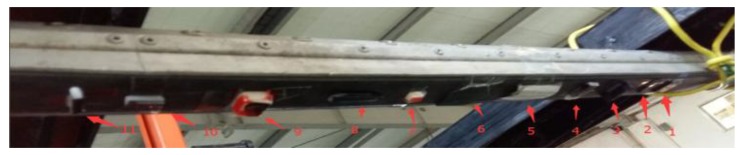
Simulated Defects.

**Figure 14:**
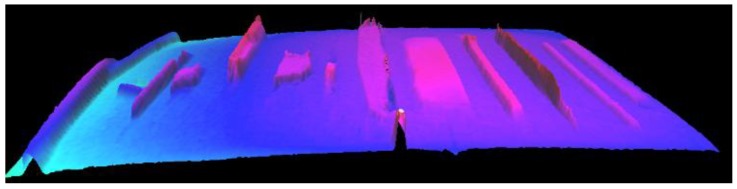
3D Results.

**Figure 15:**
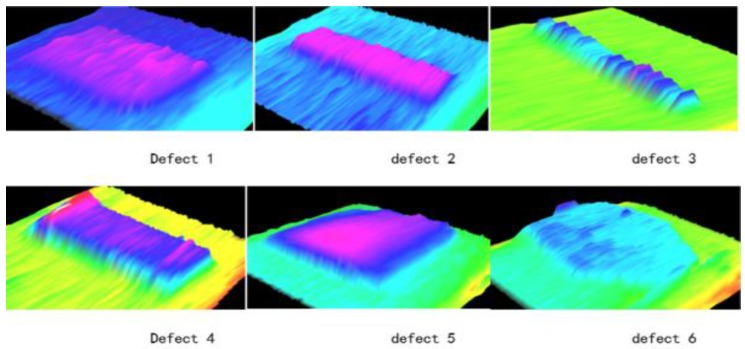

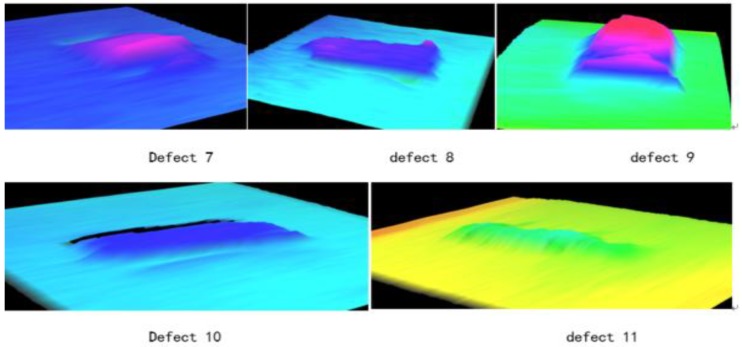
All Defects.

In order to further verify the accuracy of the layout, many defects in the laboratory environment were simulated, as shown in Figure 13. The pantograph 3D data is presented in Figure 14 and all the defects detected by the algorithm are shown in Figure 15. Table 1 shows the errors between the measured values and the actual values. The experimental results indicate that the system is featured with high accuracy and can meet the requirements of railway safety detection.

**Figure 16:**
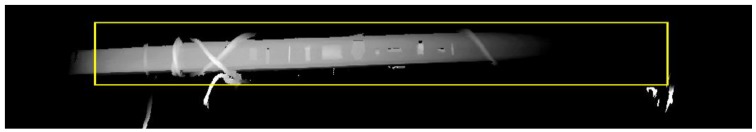
Strength images.

By mapping 3D data onto two-dimensional images, a strength image is formed, as shown in Figure 16. Then, a gradient operation is performed on the whole image to get the edge information of the defect to screen out the possible slide block area. Then, in view of the possible candidate areas, the location of the actual skateboard falling blocks is selected according to the size of the area and the height difference between the inside and outside of the area. Because the standard thickness of the patch is simulated in the experiment, the thickness of each defect is calculated by calculating the average value of each real area, as shown in Table 1.

The gradient of the image function f(x,y) in the point (*x*, *y*) is a vector with the size and direction, which is set to $G_{x}$ and $G_{y}$ to represent the gradient of the *X* direction and the *Y* direction respectively. The vector of this gradient can be expressed as:

(4) $$\nabla f\left(x,y\right)={\left[G_{x},G_{y}\right]}^{T}={\left[\frac{\partial f}{\partial x},\frac{\partial f}{\partial y}\right]}^{T}$$

The magnitude of the gradient can be expressed as Formula (5) and the direction angle of the gradient can be show as Formula (6).

(5) $$\mathrm{mag}\left(\nabla f\right)=g\left(x,y\right)=\sqrt{\frac{\partial^{2}f}{\partial x^{2}}+\frac{\partial^{2}f}{\partial y^{2}}}$$

(6) ∅(x,y)=arctan|∂f∂y∂f∂x|

According to Table 1, the root means square error (RMSE) of all data is 0.249 mm. It shows good precision. As shown in Table 1, the measured results of the same height are different. This is due to the fact that the material of the object is different and the surface reflectivity is different, leading to different intensity of light entering the sensor. The 3D reconstruction algorithm depends on the light intensity entering the sensor, so sometimes the restoration effect may be poor when it comes to the interference of strong sunlight or strong surface reflectivity. This is also a direction that can be improved in the future.

It is clearly pointed out in the operation and maintenance regulations of the EMU, published by China Railway Corporation, that the terms of pantograph detection are: (a) The skateboard cannot have a certain depth of grooves; (b) If the skateboard is damaged, the damage width must be less than 1/2 of the width of the slider. It can be seen from the maintenance regulation that there is no mature equipment in China for detecting the pantograph slide block at present and there is no quantitative index for detection accuracy as well. The experimental layout presented in this study shows a detection accuracy better than 0.5 mm. From the pantograph 3D data, it is easy to identify the pantograph slide block, so as to achieve the purpose of detecting pantograph defects.

## 7. Conclusions

This study, on the basis of the 3D camera, adopted machine vision technology for pantograph slide 3D sensor measurement through 3D data to determine whether the block of the slide is falling, which is important for railway transportation. All key parameters for the 3D detection system have been theoretically analyzed and experimentally verified. Through the artificially simulated slide surface defects, the 3D measurement system is adopted to conduct inspection.

The experimental results indicate that this method can effectively detect the skateboard impellers. Meanwhile, the three-dimensional display effect performs better than the good two-dimensional rendering display effect and 3D sensor data can obtain skateboard depth information in the full range and improve detection accuracy.

## Figures and Tables

**Table 1:** Error Analysis.

	**Defect 1**	**Defect 2**	**Defect 3**	**Defect 4**
Actual value (mm)	1.9	1.9	8.5	4.1
Detection value (mm)	2.05	2.4	8.7	4.3
Error	+0.15	+0.5	+0.2	+0.2
	**Defect 5**	**Defect 6**	**Defect 7**	**Defect 8**
Actual value (mm)	3.8	1.9	4.1	4
Detection value (mm)	3.9	2.1	4	3.6
Error	+0.1	+0.2	−0.1	−0.4
	**Defect 9**	**Defect 10**	**Defect 11**	
Actual value (mm)	9.2	6	6	
Detection value (mm)	9.5	6.1	6.1	
Error	+0.3	+0.1	+0.1	
